# Transcriptional coactivator MED1 in the interface of anti-estrogen and anti-HER2 therapeutic resistance

**DOI:** 10.20517/cdr.2022.33

**Published:** 2022-06-01

**Authors:** Gregory Bick, Jasmine Zhang, Elyse E. Lower, Xiaoting Zhang

**Affiliations:** ^1^Department of Cancer Biology, Vontz Center for Molecular Studies, University of Cincinnati College of Medicine, Cincinnati, OH 45267, USA.; ^2^Department of Internal Medicine, University of Cincinnati College of Medicine, Cincinnati, OH 45267, USA.; ^3^University of Cincinnati Cancer Center, University of Cincinnati College of Medicine, Cincinnati, OH 45267, USA.

**Keywords:** MED1, transcription cofactor, estrogen receptor, HER2, therapy resistance, RNA nanotechnology

## Abstract

Breast cancer is one of the most common cancer and leading causes of death in women in the United States and Worldwide. About 90% of breast cancers belong to ER+ or HER2+ subtypes and are driven by key breast cancer genes Estrogen Receptor and HER2, respectively. Despite the advances in anti-estrogen (endocrine) and anti-HER2 therapies for the treatment of these breast cancer subtypes, unwanted side effects, frequent recurrence and resistance to these treatments remain major clinical challenges. Recent studies have identified ER coactivator MED1 as a key mediator of ER functions and anti-estrogen treatment resistance. Interestingly, MED1 is also coamplified with HER2 and activated by the HER2 signaling cascade, and plays critical roles in HER2-mediated tumorigenesis and response to anti-HER2 treatment as well. Thus, MED1 represents a novel crosstalk point of the HER2 and ER pathways and a highly promising new therapeutic target for ER+ and HER2+ breast cancer treatment. In this review, we will discuss the recent progress on the role of this key ER/HER2 downstream effector MED1 in breast cancer therapy resistance and our development of an innovative RNA nanotechnology-based approach to target MED1 for potential future breast cancer therapy to overcome treatment resistance.

## INTRODUCTION 

Breast cancer remains one of the most common malignancies in women and is responsible for over 287,000 new diagnoses and 43,000 deaths annually in the US despite advances in treatment and detection. Based on the presence of key breast cancer biomarkers and global gene expression analyses, breast cancers can be generally subdivided into five subtypes, Estrogen Receptor (ER)+ Luminal A, Luminal B, HER2-enriched, Basal-like and the more recently identified Claudin Low subtype^[1,2]^. ER is the first and most prevalent breast cancer biomarker that is expressed at various levels in about 70%-75% of breast cancers, while the transmembrane receptor tyrosine kinase HER2 (Neu, ERBB2) is highly expressed, typically as a result of amplification of the genomic DNA containing *HER2 *and several other genes, in around 15%-20% of human breast cancer^[3-5]^. ER+ and HER2+ breast cancer subtypes thus form the vast majority of breast cancer, and anti-estrogen and anti-HER2 targeted therapies have been developed and widely used in clinics for their treatment, respectively^[6-8]^. Although the development of these ER and HER2-targeted therapies has greatly improved the patient outcome and survival, unfortunately, unwanted side effects, frequent recurrence and resistance to these treatments remain significant clinical issues and severely hinder their effectiveness^[3,9-11]^. Thus, further understanding of molecular mechanisms underlying anti-estrogen and anti-HER2 treatment resistance and the development of new targeted therapies for better and safer patient care are urgently needed. 

## CURRENT ANTI-ESTROGEN AND ANTI-HER2 TREATMENT AND RESISTANCE MECHANISMS

Anti-estrogen (endocrine) therapies include the following types based on their mechanism of action: Selective Estrogen Receptor Modifiers (SERMs), Aromatase Inhibitors (AIs), and Selective Estrogen Receptor Degraders (SERDs)^[12-14]^. SERMs, such as tamoxifen and raloxifene, compete with endogenous estrogen for the hormone binding site on the estrogen receptor and preferentially recruit co-repressors to inhibit the transcription of ER target genes^[8,15]^. AIs, such as letrozole, anastrozole, and exemestane, function by inhibiting the aromatase enzyme essential for estrogen production in the body, thus depleting the hormone required for cancer cell growth^[9,16]^. SERDs, most notably fulvestrant and several others currently in clinical trials, provide some additional benefits by targeting ER for degradation upon binding to the ER^[14,17]^. While these drugs have greatly benefited and improved the outcome for many patients, the unwanted side effects, including increased risk for osteoporosis, arthralgia, rheumatoid arthritis, and endometrial cancer, hindered their usage and effectiveness. Furthermore, the de novo (intrinsic) or acquired resistance to these treatments is highly frequent and estimated to occur in about 50% of ER+ breast cancer patients.

It is now recognized that the resistance mechanisms for anti-estrogen therapies are complex and include genetic and epigenetic alterations of growth signaling pathways, ER itself and its coregulators and pioneer factors, intracellular heterogeneity, and interactions with the tumor microenvironment and immune system, *etc.*^[18]^. The expression of ER in most endocrine-resistant tumors suggests a continued but altered role of ER, commonly through crosstalk with aberrant upregulation and activation of growth factor receptor tyrosine kinases (RTKs) and intracellular cascades such as EGFR, HER2, Insulin-like Growth Factor 1 (IGF-1), FGFR, PI3K/AKT/mTOR, RAS/RAF/MEK/ERK, CDK4/6, *etc.*^[19-25]^. Indeed, several therapies have recently been developed and approved to target these pathways (e.g., PI3K inhibitor Alpelisib, mTOR inhibitor Everolimus, CDK4/6 inhibitors Palbociclib, Ribociclib, and Abemaciclib) for breast cancer patients^[26-30]^. However, these treatments only showed limited benefit in certain patient populations and are again met with treatment discontinuation due to side effects and quick development of treatment resistance^[26,31-33]^. Thus, additional mechanistic and clinical investigations into the causes and molecular mechanisms of endocrine resistance and the development of new, safer and more effective treatment strategies are still in great need despite the success and progress of anti-estrogen treatments^[18]^. 

HER2 (Neu) is a member of the EGFR family of receptor tyrosine kinases that activate and potentiate pro-growth signaling cascades. Current anti-HER2 therapies primarily include small molecules targeting the intracellular kinase domain and monoclonal antibodies targeting the extracellular domains^[4,34,35]^. The small molecule HER2 tyrosine kinase inhibitors include lapatinib, neratinib, and tucatinib, which have been shown to inhibit cell proliferation, amplify the response to chemotherapy and anti-estrogen therapy, and potentially help limit brain metastases^[36-38]^. Despite these benefits, adverse events such as diarrhea, nausea, vomiting, and hand-foot syndrome have been documented, which may lead to treatment discontinuation ^[36]^. Monoclonal antibodies against HER2 include trastuzumab, pertuzumab, and more recently antibody-drug conjugates such as T-DM1 or T-DXd, which have trastuzumab antibodies conjugated to either the microtubule inhibitor emtansine or the topoisomerase inhibitor deruxtecan, respectively^[39-41]^. Unfortunately, targeted HER2 therapies come with their own side effect profile, too, including diarrhea, skin rash, and problematic levels of cardiotoxicity^[42-44]^. The overall response to anti-HER2 antibody therapies is limited as well, with only around one-third of HER2+ breast cancer patients ultimately benefiting from anti-HER2 antibody therapies^[36]^. Again, anti-HER2 therapy resistance and recurrence are also common, especially for patients with advanced and metastatic cancers, through point mutations affecting the ATP-catalytic site, alternate splicing/cleavage, and upregulation of other RTKs (e.g., HER3, IGF-1R, PI3K/Akt, and MET), *etc.*^[10,45-48]^. 

## ESTROGEN RECEPTOR COACTIVATOR MED1 AND BREAST CANCER

**Figure 1:**
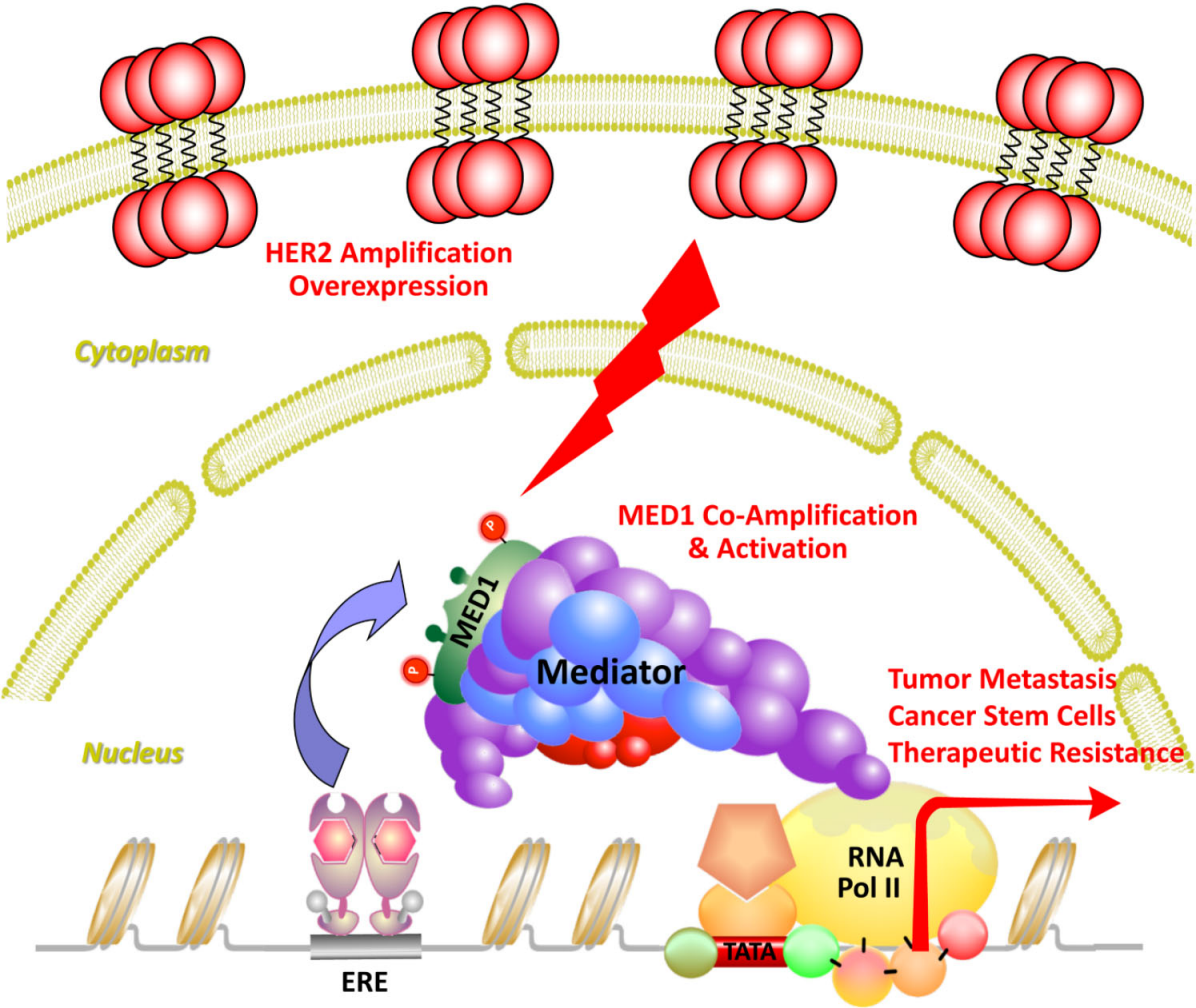
Transcription coactivator MED1 as a central crosstalk point of ER and HER2 pathways. Transcriptional activator MED1 directly interacts with ER to bridge it with RNA polymerase II and transcriptional machinery to initiate gene transcription and mediate ER functions in breast cancer. MED1 also coamplifies with HER2 and is activated by HER2 signaling cascades through phosphorylation to promote breast cancer growth, metastasis, stem cell expansion, and therapeutic resistance. Adapted from Yang *et al.*^[55]^ Cell Reports.

Estrogen receptor is a member of the nuclear receptor transcription factor family that drives breast cancer through the transcription activation of its target genes. Upon binding to hormonal estrogen, ER binds to estrogen responsive elements and promotes gene transcription through stepwise and cyclic recruitment of diverse transcriptional coactivators^[49]^. These coactivators play a variety of roles in the transcription process, such as remodeling chromatin structure (e.g., SWI/SNF), modifying histones (p300, SRCs, PRMT1, CARM1), and recruiting additional proteins or RNAs to create a permissive environment for the assembly of transcription machinery^[50-54]^. However, to initiate transcription, it still needs another coactivator complex to bridge ER and transcription machinery that is known as the Mediator [Figure 1, adapted from Ref.^[55]^]. The Mediator complex is a complex of 25-30 subunits, and mediator subunit 1 (MED1) was found to be essential for estrogen-dependent interactions between mediator complex and estrogen receptor^[56,57]^. In addition to bridging ER with RNA polymerase II and transcription machinery, recent studies also support that MED1 could participate in ER-mediated gene transcription through additional mechanisms including chromosome looping, enhancer RNA transcription, liquid-liquid phase separation and formation of transcription condensates and super-enhancers, *etc.*^[58-66]^. Recent structural studies using crystallography, cryoelectron-microscopy and cross-linking mass spectrometry have revealed detailed Mediator interactions with transcription factors and general transcription machinery in transcription initiation and Pol II phosphorylation, *etc.*^[67-70]^. Future further super-resolution structural analyses of human MED1/Mediator complex with ER, other transcriptional coregulators, and even chromatin and RNAs using these and more advanced technologies will likely provide new deep insights into the regulation and diverse functions of MED1 and Mediator in these processes. 

In breast cancer, it was found that MED1 is overexpressed in approximately 50% of breast cancers^[71,72]^. Further, MED1 is located in the genomic region containing HER2, commonly called the HER2 amplicon^[73,74]^. As such, MED1 coamplifies with HER2, and its expression is strongly correlated with HER2 amplification and expression in breast cancers. Interestingly, MED1 only exists in a subpopulation of mediator complex with a distinct subunit composition and enrichment of RNA polymerase II^[75]^. Furthermore, the MED1/Mediator complex is selectively recruited to ER target gene promoter by estrogen induction but not to other transcription factor regulated target genes (e.g., UV activation of p53), indicating some specificity of MED1 for ER-mediated transcription function. Importantly, loss of MED1 resulted in a severe abrogation of ER target gene expression in *in vitro *transcription assays, and estrogen-dependent endogenous gene transcription and growth of breast cancer cells^[56,75]^. More recently, our studies further support the key *in vivo* role of MED1 in mediating selective ER functions in pubertal mammary gland development, tumor growth, metastasis, cancer stem cell formation and therapy resistance during mammary tumorigenesis as detailed in the next few sections. 

## TISSUE-SPECIFIC ROLE OF MED1 AND ITS LxxLL MOTIFS *IN VIVO*

Estrogen receptor interacts with a variety of transcription cofactors including MED1, SRC1, and SRC3 *etc.*, via a domain on these proteins called the LxxLL motifs or NR-boxes^[76-78]^. MED1 contains two closely associated LxxLL motifs, which, when mutated to LxxAA, prevent MED1 from binding to ER in a ligand-dependent manner^[79]^. To better understand the physiological role the MED1/ER plays, a knock-in mutant mouse model was generated in which the two LxxLL motifs of MED1 were both mutated to LxxAA to inhibit MED1’s interaction with ER^[80]^. Interestingly, when the LxxLL motifs of MED1 were mutated, mice were born at appropriate Mendelian ratios, and both the heterozygous and homozygous mutants were generally healthy and fertile. The primary phenotype was in the mammary glands of homozygous MED1-LxxAA mutant knockin mice during the pubertal development. These mice showed a significant delay in mammary gland development as measured by the mammary ductal branch morphogenesis in both ductal length and number of branches^[80]^. On a cellular level, it was found that MED1 was primarily expressed in the luminal but not basal cells of the mammary gland. Significantly, it was found that the luminal progenitor cells were significantly decreased in MED1 mutant knockin mice despite MED1 being expressed at equal levels. Importantly, while total estrogen levels and estrogen-dependent uterine growth were unaffected by the MED1 mutations, the mammary gland ductal growth in response to estrogen was greatly blocked as measured by growth and ER target gene expression. 

When these MED1-LxxLL mutant knockin mice were further crossed with a HER2-driven mammary tumor model (MMTV-HER2), it was found that MED1 LxxLL mutations were able to significantly delay the tumor onset, growth and metastasis^[81]^. When compared to WT tumors, tumors from MED1 mutant knockin mice crossed with MMTV-HER2 mice showed impaired estrogen responsiveness and had much lower growth rates and metastasis. It was found that MED1 LxxLL motifs mutations significantly decreased cell proliferation, angiogenesis and cancer stem cell formation of MMTV-HER2 tumors. Consistent with these findings, the expression of a number of key ER/HER2 downstream genes involved in these processes (e.g., IGF-1, Cyclin-D1, LIF, ACP6, Twist, MMP9, VEGFA) were significantly inhibited by the MED1 mutations. Interestingly, we found that MED1 acts by directly regulating the ER downstream IGF-1 but not amphiregulin signaling pathway in our rescue experiments. This was further supported by several other findings including marked reductions in phosphorylation of IGF-1 signaling pathway proteins including the IGF-1 receptor, AKT, and mTOR in MED1 mutant tumors, phenocopying of IGF-1R inhibition and MED1 mutations in both mouse and human cancer cells, and correlation of MED1 and IGF-1 protein levels in human clinical samples. Overall, these studies support that MED1 and its LxxLL motifs play a central role in mammary tumorigenesis and suggest that MED1 could potentially serve as a tissue-specific target in breast cancer therapy.

## ROLE OF MED1 IN ANTI-ESTROGEN TREATMENT RESISTANCE

As discussed above, MED1 overexpression is a frequent occurrence in approximately 50% of all breast cancers. Interestingly, the MED1 gene is located in the HER2 amplicon and coamplifies with HER2 in almost all instances. We have further confirmed that MED1 protein levels highly correlate with HER2 status of human breast cancer by using a human breast cancer tissue microarray^[82]^. Given HER2 amplification and overexpression is a common mechanism of endocrine resistance, Cui *et al.*^[82] ^carried out to investigate whether HER2 and MED1 cooperate in driving endocrine resistance. Interestingly, it was found that MED1 levels and especially the phosphorylated form of MED1 are increased in tamoxifen-resistant BT474, MCF-7/TAM, MCF-7/HER2 cells compared to MCF-7 controls, in a manner dependent on HER2 and MAPK activation. Importantly, knockdown of MED1 in these cells was found to increase the sensitivity of these otherwise resistant cells to tamoxifen treatment. In addition to reduced expression of traditional ER target genes (e.g., TFF1, Cyclin D1) by MED1 knockdown in these cells, it was found that MED1 is also required for the expression of another class of newly defined HER2 activated ER-target genes like *LIF *and *ACP6*^[82,83]^*. *

**Figure 2:**
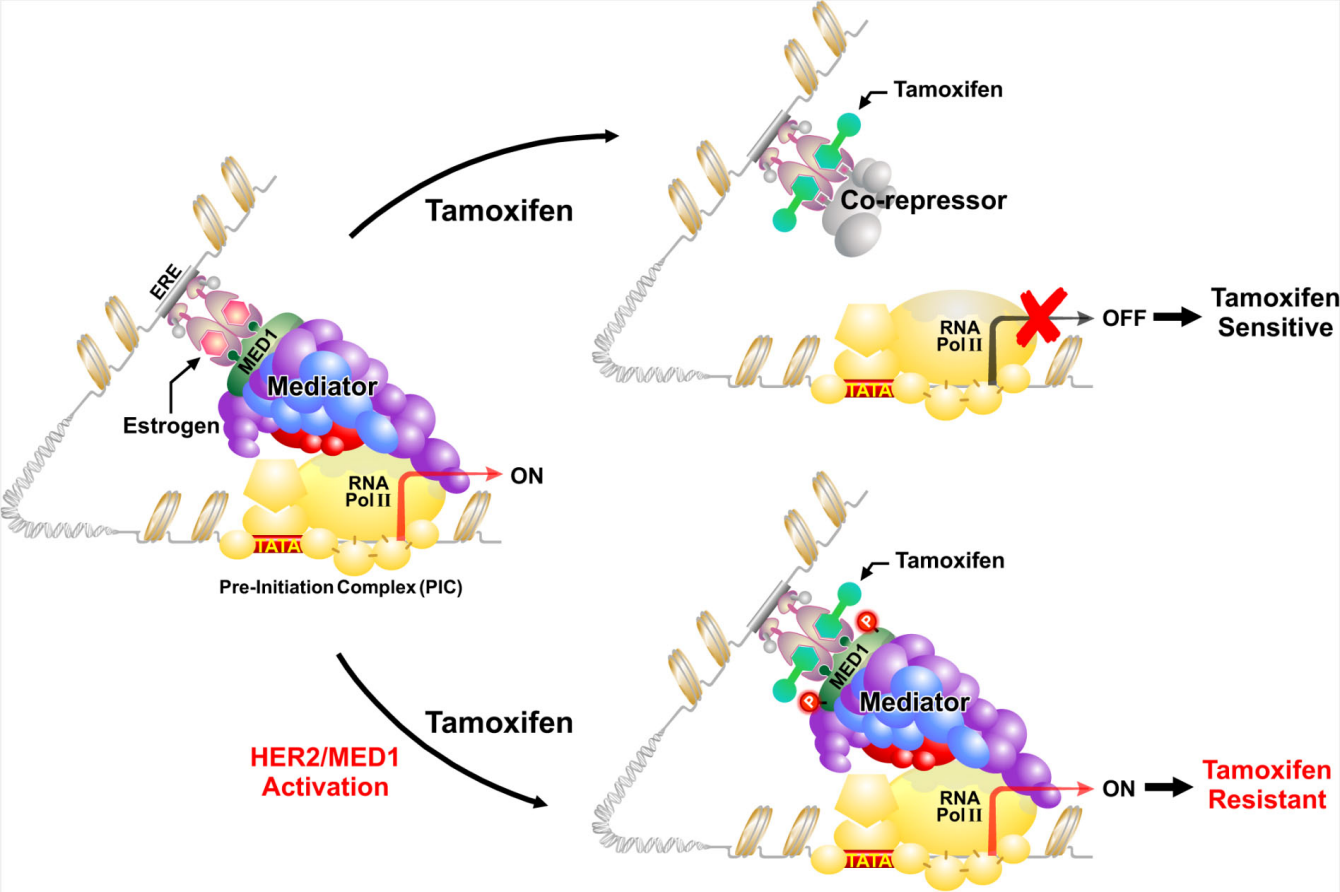
Molecular mechanisms of HER2 and MED1 in anti-estrogen treatment resistance. In endocrine-sensitive breast cancer cells, treatment with anti-estrogen tamoxifen results in the recruitment of co-repressors to suppress gene transcription. However, when MED1 is overexpressed and phosphorylated by growth factor cascades such as HER2, the MED1/Mediator complex rather than ER co-repressors is recruited to activate gene transcription and render endocrine resistance. Adapted from Leonard *et al.*^[72]^, JZUS-B.

Mechanistically, it is known that when tamoxifen binds to ER, the conformational change forces ER into a repressive state, and rather than recruiting coactivators, ER preferentially recruits co-repressors such as NCoR and SMRT to inhibit transcription of downstream target genes^[15]^. However, in these tamoxifen-resistant cells with MED1 overexpression and activation, it was found that tamoxifen treatment still resulted in the recruitment of MED1 and phospho-MED1 but not co-repressors to the ER target gene promoters [Figure 2]. Importantly, this was completely reversed with MED1 knockdown in these cells and restored the recruitment of the co-repressors under tamoxifen treatment. Furthermore, it was found that only wild-type but not phospho-defective mutant of MED1 (T -> A) was able to displace the co-repressors after tamoxifen treatment in these MED1 knockdown cells, supporting the importance of MED1 phosphorylation by HER2 in mediating tamoxifen resistance.

Consistent with our studies above, other laboratories have also reported clinical evidence indicating MED1 as a gene associated with poor endocrine treatment outcome, and that high MED1 expression correlates with poor survival of breast cancer patients that have undergone anti-estrogen therapy^[84,85]^. In addition, another genome-wide study identified an increased frequency of MED1 mutations in circulating plasma DNA in breast cancer patients following anti-estrogen and anti-HER2 treatments^[86]^. These studies suggest a broad role of MED1 in anti-estrogen treatment resistance to other anti-estrogen therapies besides tamoxifen. Indeed, we found that MED1 depletion sensitized the MCF-7 cell derived fulvestrant resistant cell line (MCF-7-F) and HER2/MED1 overexpressing BT-474 and ZR-75.1 cells to fulvestrant treatment as well *in vitro* and *in vivo*^[87]^. Together, these data support that MED1 overexpression and activation by growth factor signaling pathways like HER2 can drive broad resistance to anti-estrogen treatments, and that MED1 could serve as a potential therapeutic target to overcome such treatment resistance.

## TARGETING MED1 IN ANTI-ESTROGEN RESISTANCE BREAST CANCER BY RNA NANOTECHNOLOGY

**Figure 3:**
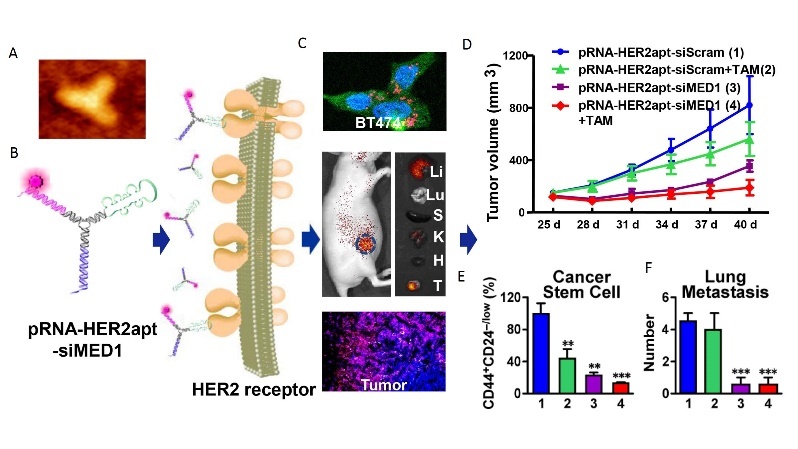
Overcoming breast cancer therapeutic resistance by MED1 targeting multifunctional RNA nanoparticles. The figure shows the atomic force microscopy (A) and schematic (B) of the pRNA-HER2apt-MED1siRNA nanoparticle, its tumor-specific uptake *in vitro and in vivo *(C), and therapeutic effect to inhibit endocrine-resistant tumor growth (D), cancer stem cell formation (E), and lung metastasis (F) *in vivo *in an orthotopic xenograft mouse model. **P < 0.01, ***P < 0.001. Adapted from Zhang *et al.*^[88]^, ACS Nano.

Based on these findings, Zhang’s laboratory has developed a targeted approach to block MED1 specifically in anti-estrogen resistant breast cancers using an innovative RNA nanotechnology approach^[88]^. The reason we selected this approach is due to many advantages of the RNA nanotechnology: multifunctional capabilities with simultaneous targeting and therapy, high stability and nanoscale size allows slower body clearance and better tissue penetration, retention, and cellular uptake, low toxicity and immunogenicity, controlled synthesis and self-assembly for great manufacturing and scale-up compatibility, *etc.*^[88-93]^. Furthermore, this technology also allows for the development of ways to target the transcriptional coactivators like MED1 that are difficult to target with traditional small molecules or antibody-based approaches. By using a phi29 3-way junction pRNA nanodelivery system, we have created RNA nanoparticles that are composed of two MED1-targeting siRNAs to silence MED1 and an aptamer that specifically binds to membrane receptor HER2 to allow for targeted delivery into breast cancer cells [Figure 3]. These pRNA-HER2apt-siMED1 RNA nanoparticles can be generated uniformly with high reproducibility as measured by dynamic light scattering and atomic force microscopy. By using 2’Fluoro modified nucleotides, these RNA nanoparticles exhibited high stability, even in conditions that mimic or are more extreme than physiological conditions, including treatment with RNase A, Serum, Urea or heat up to 70 degrees Celsius. Importantly, we have shown that treatment with recombinant Dicer enzyme can easily release the siRNA arms from the nanoparticle, an essential step in the RNAi pathway.

These highly stable multifunctional RNA nanoparticles have been successfully tested in both *in vitro *and *in vivo *preclinical models and exhibited highly desirable tumor-specific targeting and treatment efficacy with no apparent toxicity^[88]^. It was found that the pRNA-HER2apt-siMED1 nanoparticles can silence MED1 expression in both de novo tamoxifen-resistant BT-474 cells that have HER2 amplification and acquired tamoxifen-resistant MCF-7/TAM cells with elevated HER2 expression but no amplification. These knockdown effects are specific and dependent on both siMED1 sequences and intact HER2 binding aptamer. Furthermore, triple-negative breast cancer MDA-MB-231 cells were not able to take up these RNA nanoparticles or knockdown MED1. In addition to growth inhibition, we found that these MED1 targeting RNA nanoparticles significantly reduce cell migration, stem cell formation, and MED1 target gene expression compared to control scramble RNA nanoparticles or tamoxifen alone. When we cotreated these cells with tamoxifen, we found further decreased cell growth, motility, and cancer stem cell formation compared to either treatment alone. 

The distribution and the efficacy of pRNA-HER2apt-siMED1 nanoparticles were further examined *in vivo *using a human breast cancer orthotopic xenograft mouse model. It was found that pRNA-HER2apt-siMED1 but not the HER2apt mutant nanoparticles were able to significantly accumulate and enrich in the tumors while both showed only residual levels in the liver and kidneys, and were undetectable in other organs such as lung, spleen and heart. Remarkably, we found that once a week systemic injection of the RNA nanoparticles through tail vein achieved greater tumor growth inhibition *in vivo *than regular 5 days per week tamoxifen treatment. Immunochemistry staining and western blot analyses confirmed that the expression of MED1 was significantly depleted in the pRNA-HER2apt-siMED1-treated groups along with a great reduction of Ki-67 expression, tumor growth, lung metastasis and stem cell formation compared to those of control treatments. Importantly, we did not observe apparent toxicity, and there was no body weight change or histological abnormalities in major organs during the course of treatment including liver and kidney. This lack of toxicity in these organs could be due to one or a combination of the rapid tissue clearance of the RNA nanoparticle, our tumor-specific HER2 aptamer ligand design and the inability of RNA nanoparticles to enter the cells in these organs, as well as the tissue-specific roles of MED1 and its lack of key biological functions in these organs, *etc. *Together, these findings support that our biosafe pRNA-HER2apt-siMED1 nanoparticle represents a highly promising new therapeutic regimen for potential future breast cancer treatment to overcome resistance. 

## MED1 IN HER2-MEDIATED TUMORIGENESIS AND ANTI-HER2 THERAPY RESPONSE

We have discussed above that MED1 is frequently overexpressed and coamplified in breast cancers with HER2 and crosstalk with HER2 in mediating anti-estrogen treatment resistance. However, it was still not known whether MED1 plays a role in mediating HER2-mediated tumorigenesis and the response to anti-HER2 treatment. To achieve that, we have generated a MED1 mammary specific overexpression mouse model MMTV-MED1^[55]^. We found that over-expression of MED1 in mammary glands resulted in a slightly increased number of mammary stem and progenitor cells, but no other phenotypical abnormalities or increase in cancer incidence. However, when we crossed these MMTV-MED1 mice with the MMTV-HER2 mammary tumor-prone mouse model, we found a strong increase in mammary tumor formation marked both by the earlier onset of an initial tumor and an increase in the number of individual tumors formed per mouse. Furthermore, these MMTV-HER2/MMTV-MED1 tumors grew much faster and formed more lung metastases compared to MMTV-HER2 tumors. These *in vivo *findings were further validated by *in vitro *assays showing that isolated MMTV-HER2/MMTV-MED1 tumor cells had higher tumor mammosphere forming ability and metastatic capabilities. Flow cytometric analysis using stem cell markers and limiting dilution assays further indicated a higher stem-like cell content in MMTV-HER2/MMTV-MED1 tumors compared to that of MMTV-HER2 tumors.

Interestingly, MED1 was found to play a role in the tumor response to anti-HER2 treatment as well. It was found that MMTV-HER2/MMTV-MED1 tumors were highly resistant to anti-HER2 lapatinib treatment, as these tumors, despite treatment with lapatinib, still grew at the same rate as vehicle-treated HER2+ tumors and significantly faster than lapatinib treated HER2+ tumors^[55]^. Furthermore, MMTV-HER2/MMTV-MED1 tumors could still readily metastasize to the lung after lapatinib treatment, while the same treatment eliminated lung metastasis of MMTV-HER2 tumors. This is consistent with high Ki67 staining and the number of tumor-initiating cells by limiting dilution assays observed in MMTV-HER2/MMTV-MED1 tumors compared to that of MMTV-HER2 tumors after the treatment. Similar phenomena were also observed using human breast cancer cells and further supported the role of MED1 in HER2-treatment responses. Additional work is ongoing to test the role of MED1 in resistance to other anti-HER2 small molecule and antibody therapies discussed above. Mechanistically, it was found that MED1 overexpression results in increased expression of a variety of downstream genes, including JAB1, which regulates protein ubiquitination. Interestingly, MED1 was found to be a target of JAB1 directed ubiquitination as well, which was found to be essential for MED1’s cyclic recruitment to its target genes’ promoters and the expression of these target genes. Importantly, it was found that MED1 and JAB1 protein levels were closely correlated, particularly in HER2 positive breast cancer clinical samples^[55]^. Together, these data indicate that MED1 plays critical roles in HER2-mediated tumorigenesis and therapy response, and MED1 therapy could be potentially utilized in the treatment of HER2+ breast cancers as well.

## CONCLUSIONS AND FUTURE PERSPECTIVES

Despite the development and advancement in targeted therapies for ER+ and HER2+ breast cancers, severe unwanted side effects, frequent resistance, and relapse remain the greatest clinical challenges. In this review, we have provided experimental and clinical evidence from our laboratory and others that support the key roles of MED1 in both anti-estrogen and anti-HER2 treatment resistance and the potential use of MED1 therapy to overcome treatment resistance. It will be important in the future to further understand the molecular mechanisms underlying MED1 functions and its upstream and downstream regulators in mediating treatment resistance, as these will not only help us better understand the basic cancer biology of these tumor subtypes but also provide potential new therapeutic targets. In ER+ breast cancers, it will be important to understand what drives MED1 overexpression, the further fundamental molecular details on how MED1 regulates ER target gene expression, and whether targeting MED1 could delay the development of anti-estrogen treatment resistance, *etc. *It will also be interesting to know further the role and molecular mechanisms of MED1 in the treatment resistance of HER2+ tumors to both anti-HER2 small molecules and antibodies, whether it plays a role in mediating the poor response of ER+ tumors in HER2+ heterogeneous tumors, and what role it might play in HER2+ER- tumors and the mechanisms, among others.

Many of these questions should be addressed in appropriate breast cancer cell lines, organoids, orthotopic xenograft, and PDX models, and most importantly, tested in clinics. Significantly, we have developed an innovative patented RNA-nanotechnology-based approach to target MED1 specifically in breast cancer cells and successfully tested it in both *in vitro* and *in vivo* preclinical models. Given the many advantages of RNA nanotechnology described above and our highly stable multifunctional RNA nanoparticles designed with specific tumor targeting and dual MED1 siRNA therapeutic modules, we expect our MED1 RNA therapeutics to be less toxic, more effective, and less prone to develop treatment resistance. Since MED1 functions far downstream in the ER and HER2 pathways at the last step before transcription initiation, targeting MED1 will not only help gain better treatment outcomes but will likely be less prone to the development of resistances commonly occurring through aberrant activation of upstream signaling kinases or mutations in ER itself. Finally, the better safety profile and long-lasting effects with fewer treatments needed for our RNA nanoparticles could also mean better patient treatment compliance as well which is also a major issue with current treatment. With increasing FDA approval, large-scale production and broad use of RNA-based vaccines and medicines, we are very excited about the opportunities and fully anticipate that our RNA nanotherapeutics could represent a highly promising next-generation therapy to ultimately benefit patient care through better efficacy, fewer side effects, and improved patient quality of life and treatment compliance. 

## DECLARATIONS

### Copyright

© The Author(s) 2022.
